# A youth advisory group on health and health research in rural Cambodia

**DOI:** 10.1080/11287462.2024.2361968

**Published:** 2024-06-07

**Authors:** Mom Ean, Rupam Tripura, Phann Sothea, Uch Savoeun, Thomas J. Peto, Sam Bunthynn, James J. Callery, Ung Soviet, Lek Dysoley, Phaik Yeong Cheah, Bipin Adhikari

**Affiliations:** a Mahidol-Oxford Tropical Medicine Research Unit, Faculty of Tropical Medicine, Mahidol University, Bangkok, Thailand; b Centre for Tropical Medicine and Global Health, Nuffield Department of Clinical Medicine, University of Oxford, Oxford, UK; c Techo Siem Pang High School, Provincial Department of Education, Youth and Sport, Siem Pang, Stung Treng, Cambodia; d Siem Pang Health Centre, Provincial Health Department, Siem Pang, Stung Treng, Cambodia; e District Governor Office, Siem Pang District Administration, Siem Pang, Stung Treng, Cambodia; f Provincial Health Department, Stung Treng, Cambodia; g CNM National Centre for Parasitology, Entomology and Malaria Control, Phnom Penh, Cambodia; h School of Public Health, National Institute of Public Health, Phnom Penh, Cambodia

**Keywords:** Youth groups, community engagement, health, research, skills, trust, relationships, theory of change

## Abstract

Engaging young people in health research has been promoted globally. We explored the outcomes of youth advisory group on health and research engagement (YAGHRE) in rural Cambodia. In May 2021, the Mahidol Oxford Tropical Medicine Research Unit (MORU) partnered with a local health centre and a secondary school to establish a youth engagement group. Ten students underwent training and led health engagement activities in schools and communities. Activities were documented as field notes and audio-visual materials which underwent content analysis using theory of change supplemented by iterative discussions with YAGHRE members and stakeholders. Five major outcomes were identified: *1. Increased respect*. Engagement activities developed based on input from students and stakeholders may have fostered greater respect. *2. Built trust and relationships*. Frequent visits to MORU’s laboratory and interactions with researchers appeared to contribute to the building of trust and relationship. *3. Improved health and research literacy*. Learning new health and research topics, through participatory activities may have improved literacy; *4. Improved uptake of health and research interventions*. Health promotional activities and communication with research participants potentially increased the uptake of interventions; *5. Improved community health*. YAGHRE’s health promotional interventions may have contributed in enhancing community’s health.

## Introduction

Community engagement (CE) for global health research has gained increasing attention in recent years (Adhikari et al., 2020; Tindana et al., 2007; Vincent, Adhikari et al., 2022). Despite a growing literature, what constitutes CE and its rationale remain heterogeneous (Adhikari et al., 2016; Adhikari, Vincent et al., 2019; Tindana et al., 2007). CE is an essential component of global health research to improve the ethics of study conduct, and participant recruitment and retention (Adhikari, Vincent et al., 2019; Adhikari et al., 2020). But viewing CE as an ancillary component (to promote research participation) of health research has been criticized as it overlooks the ethical goals ascribed to it (Adhikari, Vincent et al., 2019; Reynolds & Sariola, 2018). Engagement activities are also conducted independent of a specific research project to serve ethical goals such as increasing health literacy, capacity-building, and bridging the gaps between researchers and the public (Davies et al., 2019; Ean et al., 2021; Lim et al., 2016; Lim et al., 2017; Nguon et al., 2018).

The literature on engaging school students highlights the process and outcomes of such engagement (Davies et al., 2020; Pol et al., 2017). Participatory engagement processes have been described as addressing the core of CE: empowering community members to respond to the topic of concern and contribute to potential solutions relevant to their local context through action and reflection, rather than the traditional approach of outsiders introducing and imposing solutions to their problems (Baum et al., 2006; Callery et al., 2021).

CE is increasingly being promoted globally, more so recently because of divisions between scientists and the public which can create two antagonistic factions in society (Adhikari et al., 2022; Goldenberg, 2016; Goldenberg, 2021). If the widening gaps between science-practitioners and the public are not redressed, there is the potential for further misunderstanding and distrust towards science (Adhikari & Cheah, 2021; Goldenberg, 2021). There are already warning signs of how some sections of the public have become sceptical of science and its products, most saliently during the COVID-19 pandemic (Rutjens et al., 2021). Over the last few years, dominated by the digital media, narratives against scientific expertise have increased, and are evidenced by the rise of alternative theories about the SARS-CoV2 virus, and false information about cures for COVID-19 disease (Freckelton QC, 2020). The impact was clear as demonstrated, for instance, by low vaccine uptake in high-income countries together with demonstrations against the government on the imposition of public health measures and vaccination (Edwards et al., 2021). An increasing number of studies illustrate this antagonism (Goldenberg, 2016; Goldenberg et al., 2023 Hornsey & Fielding, 2017;). Left as it is, this can have serious consequences, for example, increased morbidity and mortality due to emerging infectious diseases among vaccine refusers who may be motivated by conspiracy theories and scepticism towards science in general (Dyer, 2021; Freckelton QC, 2020; Goldenberg, 2021).

Investment in promoting scientific literacy through lay people by engagement is a potentially important part of the solution to mitigate the public-science gap in trust (Adhikari, Hlaing et al., 2019). Efforts towards reducing the gaps are being implemented by major research institutions that include translating research and science to the lay public such as through public events (Adhikari, Hlaing et al., 2019). Public events are now often promoted by academic institutions where scientists are encouraged to engage with the public, share their knowledge, rectify the misconceptions around science and ultimately increase the trust and relationship with the public who are the end-users of science and its outcomes (Robinson et al., 2017).

One important method to connect with the public is through the young community members who can form a bridge between the public and the scientists; and can become agents of social change (Ho et al., 2015; Pontón & Andrade, 2007). Apart from youth members themselves being the end-user of science, they can also be a conduit between scientists and the wider community members because of their enthusiasm to learn and share new knowledge (Ho et al., 2015; Pontón & Andrade, 2007). The United Nations Convention on the Rights of the Child (CRC) enshrines the participation of children in evidence generation and policymaking as essential and outlines the obligation of a state to create an enabling environment that allows the views of children and adolescents to be heard on practices and policies that directly and indirectly concern them (UNICEF, 2021). The United Nations Education Scientific and Cultural Organization (UNESCO) has been engaging youth groups since 1999 to discuss the development of their communities, foster peace, and alleviate poverty and inequality (UNESCO, 2021).

The literature around engagement has shown how engaging school students who have a brief orientation to science in school can have manifold positive consequences (Davies et al., 2019, 2020). In this article, we describe the establishment of a youth advisory group on health research and engagement (YAGHRE) in north-eastern Cambodia, their activities to date and outcomes achieved one year on.

## Methods

### Setting

**Figure 1.**
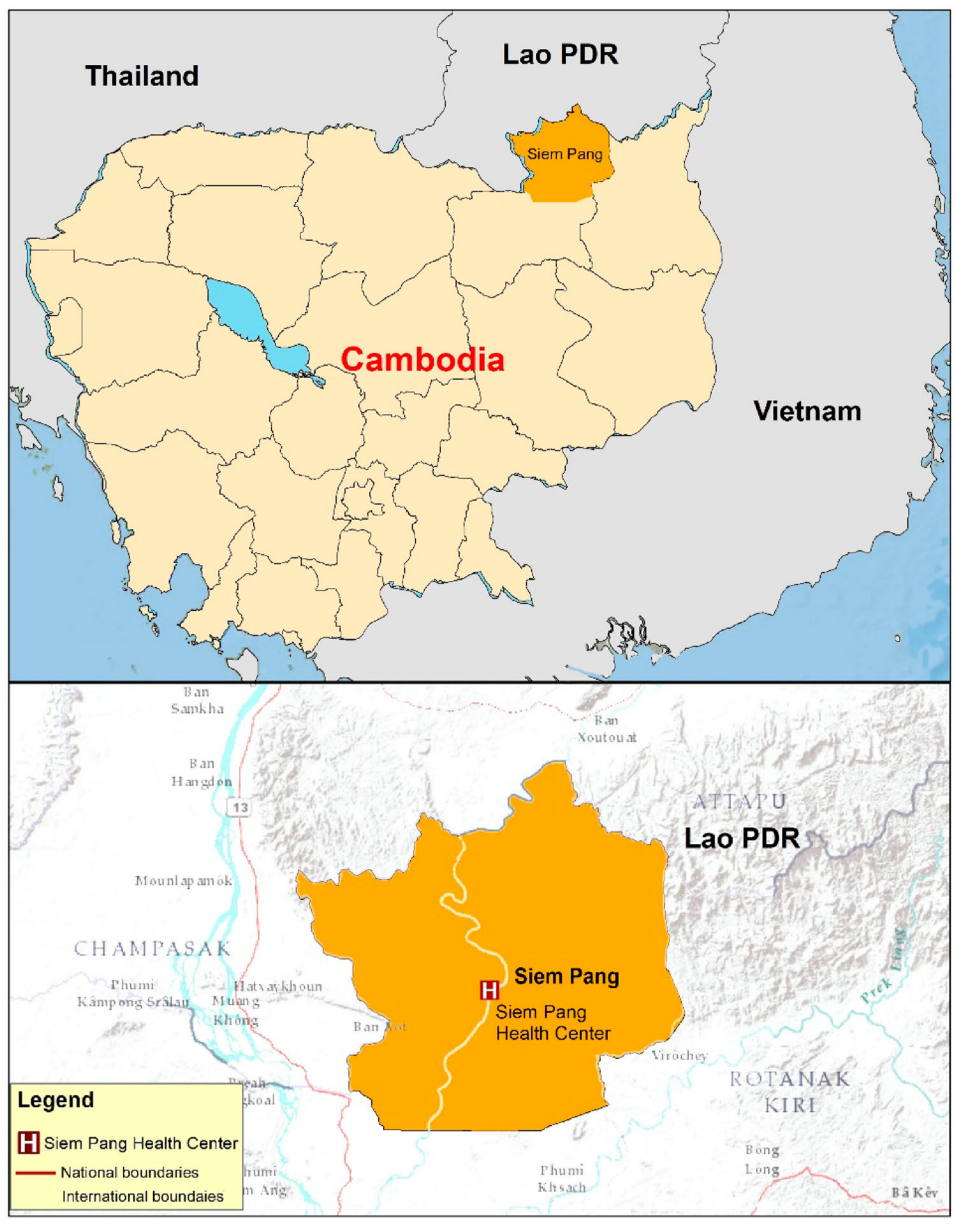
MORU’s research site, Siem Pang, north-eastern Cambodia.

The Cambodian National Malaria Control Program (CNM, an entity within the Ministry of Health) and the Mahidol Oxford Tropical Medicine Research Unit (MORU, an academic institution) have been collaborating to conduct a wide spectrum of health research that ranges from qualitative studies to multi-country clinical trials, focused primarily on malaria since 2007. The YAGHRE was established in May 2021 as an integral component of the activities at a research station located within the health centre in Siem Pang, a district located in Stung Treng Province in north-eastern Cambodia (Figure 1) where MORU has been conducting studies since 2017. The YAGHRE was also designed to align with MORU’s overall engagement strategies that outline three interrelated broad outcomes: 1. MORU programmes are trustworthy; 2. The potential health impacts of MORU programmes are maximized; and 3. MORU programmes are ethical (MORU, 2019). To achieve these outcomes, MORU conducts a wide range of engagement activities that include (community) advisory groups, participatory science–arts collaborations, festivals and themed events, public talks, stakeholder engagement workshops, and dialogues with community leaders, participants and communities (MORU, 2019). The YAGHRE is one of five community advisory boards facilitated by MORU, the oldest one being the Tak Province Community Ethics Advisory Board (Cheah et al., 2010).

There are 37 public schools in Siem Pang district which has a total population of around 25,000. A total of 66% of the children population attend school and of which around 79% graduate from high schools annually. In Cambodia, public schools start with grade one (starting age 6–7 years) that teaches the basics of education (such as mathematics, and the Khmer alphabets) and progressive grades end at grade 12 after which students graduate high school. Students are aged between 18 and 22 years at graduation from high school. Formal education in public schools in Cambodia entails studies of major subjects that include Mathematics, English, Khmer language, Biology, History, Physics, Chemistry, Morality, Geography, and Physical Education. Apart from these subjects, students also involve themselves in extra-curricular activities such as participating in quizzes, essay competitions, and sports. Nonetheless, these students have limited opportunities to study science and research, or to develop soft skills such as those related to the communication of knowledge, public speaking, and preparing presentations.

At the time of inception, engagement with the YAGHRE was aimed to 1. embed YAGHRE members’ voices in research, 2. co-create health- and research-related information for the lay public, 3. improve the quality of health in the community through health education, and 4. identify and address the issues and concerns surrounding research activities and participants.

### Youth group

**Figure 2.**
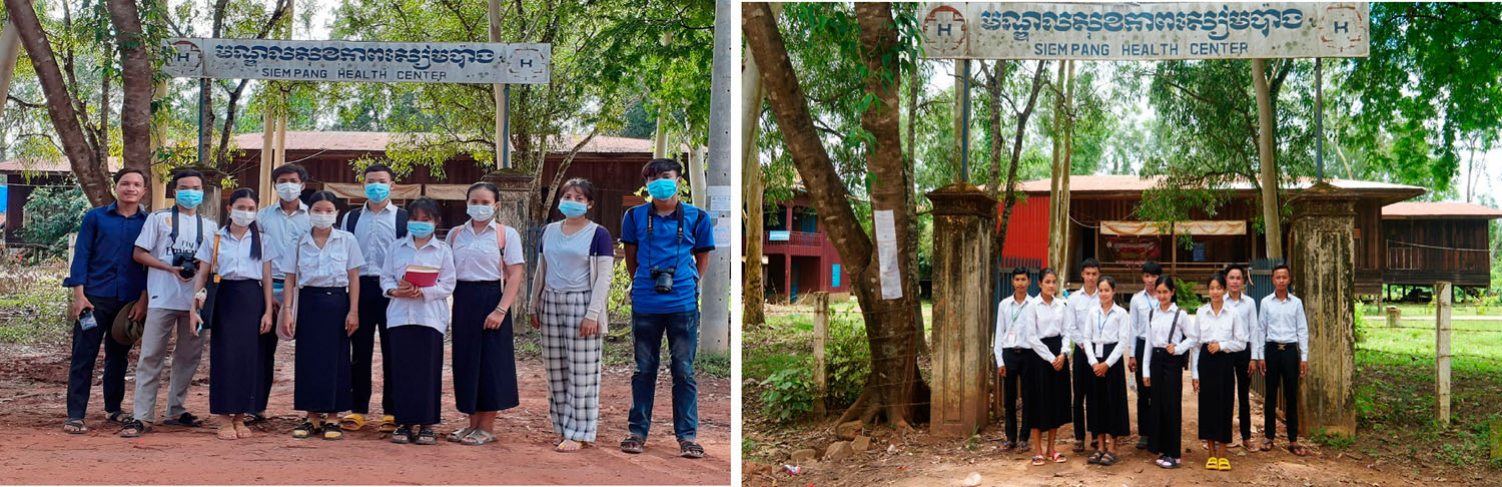
Photo of YAGHRE members.

The YAGHRE refers to a cohort of an initial 10 students (Figure 2) who were selected by the school principal and the class teacher in discussion with MORU’s public engagement team based on their interest in participating in youth engagement activities. All of these students volunteered to join the group and participate in the activities. They were students from grade 11 to grade 12, between 18 and 22 years old who had interests in extra-curricular activities, such as learning and contributing to science and research. These students dedicated additional hours after their school to participate in YAGHRE activities.

### Reflexivity

The YAGHRE in Siem Pang is led by ME who is a Khmer woman and, a native of Siem Pang. All data and reports were recorded by ME and her field staff together with YAGHRE members. RT and BA are MORU research physicians and engagement supervisors (non-Khmer) who closely supported the engagement activities (MORU, 2019). Our initial reflective exercise centred around how the activities were serving the initial aims of YAGHRE, but we later analysed the engagement activities’ outputs and outcomes using the theory of change approach. Considering the concept of reflectivity outlined by prior literature (Ben-Ari & Enosh, 2011; Jasper, 2005; Mortari, 2015), our reflection entailed an active, iterative, and theory-driven knowledge building approach to explore whether our activities support or refute goals ascribed to CE for ethical global health research (Adhikari et al., 2017). Nonetheless, our reflectivity is constrained by our position as embedded researchers, while at the same time, reflectivity may not be possible for a detached researcher from the event/phenomenon. The findings therefore retain our interpretative analysis of the activities engaging with the broad goals of CE.

### Data collection and analysis

**Figure 3.**
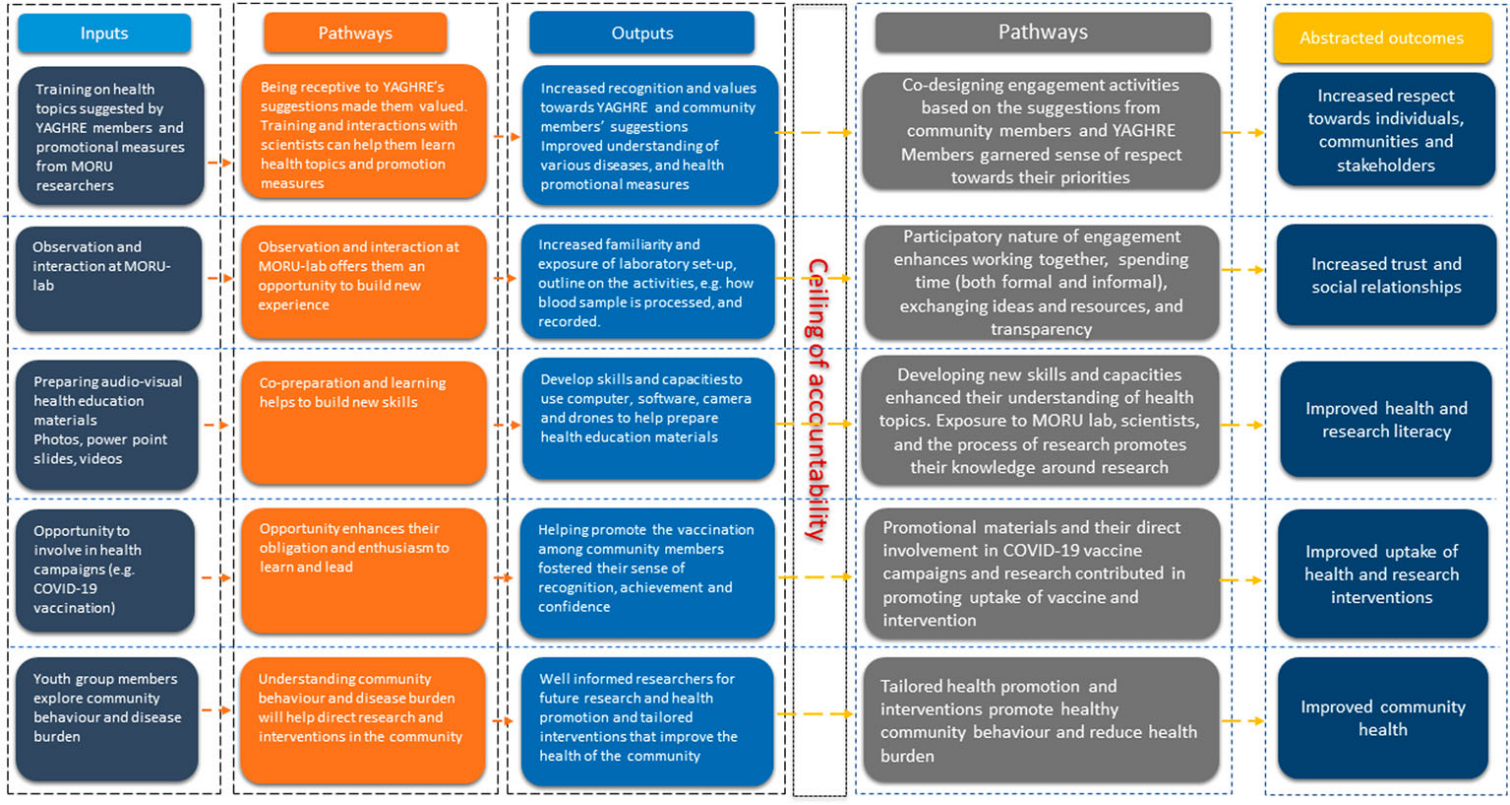
YAGHRE theory of change framework.

Each engagement activity was recorded in meeting minutes in the form of fieldnotes, photographs, and videos. The team overseeing the engagement (ME, RT, and BA) and engagement staff met at least monthly (and also on an ad hoc basis) to reflect on the activities, overarching objectives, and ways to meet the objectives and processes. In addition to the reports arising from regular reflection and planning sessions, further meetings and discussions with YAGHRE members as an annual review were conducted which helped to consolidate our objectives, activities, and potential outcomes (Table 1). Ultimately, all meeting reports, resources used in engagement and overarching objectives and goals underwent reiterative process of content analysis using the theory of change approach to synthesize outputs and outcomes. The components (inputs, pathways, outputs, and outcomes) within the theory of change were discussed and refined after discussing with the YAGHRE members. The theory of change approach outlines a stepwise process of evaluating “input” which refers to what has been invested (e.g. training, and resources); pathways (e.g. mechanism that led to achieve the intended output) and outcomes (e.g. changes in certain behaviour, or development of certain skill sets) (De Silva, Breuer et al., 2014). The initially drafted theory of change was further refined in a workshop in Bangkok where engagement experts and external evaluation consultants provided comments and suggestions (Figure 3).

**Table 1.** Brief description of data, objectives and potential outcomes of school engagement activities in Siem Pang.

Activities	Objectives	Outcomes
Orientation meeting	Orientate all partners about the youth engagement activities. Agree plans for implementation. Clarify roles and responsibilities Conduct training workshops for YAGHRE members.	– Organized orientation meeting with the Siem Pang deputy governor, chief of district education office, chief of both health centres, school principal, teachers, and students – All stakeholders were informed about the project, agreed on all project activities including opportunities and responsibilities – Selected 10 students from grade 11 (5 female and 5 male), who were enthusiastic, competent, and willing to take the opportunity – We received two batches of students as YAGHRE members (until the time of this report) – YAGHRE members were subsequently trained in their role – Increased awareness about MORU and its research among YAGHRE members and stakeholders – The project received support and appreciation by the Siem Pang governors and – Participation by all stakeholders
YAGHR members Learn about MORU	Increase understanding about MORU’s history, and its research processes among YAGHRE members and community. Promote relationship and trust between community members and MORU. Increase awareness about MORU’s research priority and future plans among YAGHRE members and stakeholders.	– YAGHRE members learned about the history of MORU, its establishment in Southeast Asia, countries where it operates, and on disease priorities. – YAGHRE members learned about MORU, its research sites in Cambodia, and studies conducted in Siem Pang since its establishment in 2017 – YAGHRE members learned about malaria, patients (signs and symptoms, and potential transmission), work process in laboratories (including malaria parasite under the microscope). – YAGHRE members learned the process of patient screening and work process in the lab during patient enrolment. – Once YAGHRE members are aware of MORU’s research projects in Siem Pang, they share their knowledge to their family, friends and community members thus promoting transparency and trust on MORU’s research team and work.
Learn to use Computer	To provide YAGHRE members with knowledge and skills on how to use the various programmes of computers. To build the capacity of YAGHRE members to use computers proficiently.	– YAGHRE members increased their knowledge and skills related to the use of computer for example they can write brief reports and minutes of meetings. – YAGHRE members had not learnt about Microsoft Power point, but they have learnt the skills to prepare slides. Apart from health education materials, they have learnt to make posters which were presented to fellow school students including presenting them in international meetings. – Members have learnt to use e-mails, following the creation of individual email addresses. They also learnt how to use Zoom, Skype, and Microsoft Team for communication and meetings, including recording for future purposes. – YAGHRE members have created a Facebook and YouTube pages for their regular communication of health educational materials.
Learn to use Drone	To build the capacity of YAGHRE members to use drones for audio-visual preparation. To record all engagement activities.	– YAGHRE members learned the detailed instructions on the function, benefits, and methods of launching a drone. – Members learnt how to operate the drone, acquire pictures and videos relevant for the educational materials. – Members learnt the drone footage processing software. Footages and pictures were compiled to produce the health educational audio-visual materials. – For instance, YAGHRE members used drone-produced audio-visual materials for COVID-19.
Learn to use camera	To enhance skills related to photography and video production. To take pictures of daily work activities.	– A total of three cameras were provided to YAGHRE members to learn and practice following the training. YAGHRE members were provided three cameras to practice photography and video making. – YAGHRE members gained knowledge and skills in using the camera for taking pictures and videos of daily engagement activities. For instance, videos of COVID-19 vaccine promotion campaigns, school engagement activities, and disease-related videos.
Learn to produce video	To build skills in building health educational materials, compiling pictures and video clips to compose comprehensive audio-visual materials.	– YAGHRE members explored intermediate video editing software on computers and mobile phones during one month of study – Members learnt to explore ideas, and work in a team to create audio-visual materials.
Participation in COVID-19 vaccination campaign	To facilitate YAGHRE members’ participation in COVID-19 vaccination campaigns.	– YAGHRE members supported the social campaigns related to health promotion. YAGHRE members participated together with a team of physicians and district authorities to promote vaccination against COVID-19. – Despite the remoteness of the communities and the limitations of health services, the district coverage of COVID-19 vaccination was 87%. – YAGHRE members learnt the process of vaccination, its delivery and population-wide implementation including filling up consent forms for vaccination and its utility. – Members received the opportunity to know and work with authorities on such social campaigns. – Members also learnt about the COVID-19 and the benefits of vaccination. – Members prepared key messages through posters and leaflets to engage with community members to promote the benefits of vaccine and population coverage. – Members supported the health care workers in preparing the vaccination for community participants (e.g. storage, delivery, counselling and organization, and support to community members). – YAGHRE members received appreciation from the government authorities (e.g. certificate of appreciation from the district government).
Health engagement at school on COVID-19	To promote solidarity to fight with COVID-19 in the community. To know what students and family members know on preventing COVID-19.	– School principal and teachers welcomed the MORU team and appreciated the health engagement program. – YAGHRE members provided key messages on COVID-19 to 159 students in 3 secondary schools in 2021-22 – School students’ knowledge related to COVID-19 was assessed (pre and post engagement) and were found to have improved the knowledge. – Knowledge related to COVID-19 was further shared among community members by the students, including YAGHRE members. – Based on the pre and post questionnaire, a total of 194 family members were found to receive COVID-19-related messages. – Members joined with the government and Ministry of Health to prevent the spread of COVID-19, to promote healthy behaviour (e.g. wearing of face mask, sanitation, and overall hygiene). – Through such participation in COVID-19-related health campaigns and vaccination, YAGHRE members learned communication, teamwork, presentation skills and leadership. – YAGHRE members learnt more about COVID-19 based on the pre and post engagement with school students and community members.

### Components of a theory of change

A theory of change is a purposeful model of how a set of activities as an engagement strategy, contributes through a chain of early and intermediate outcomes, pathways, and assumptions to the intended result and helps to navigate the complexity of social change (Serrat, 2017). Reflecting on our engagement activities, we categorized engagement activities into five major inputs into the theory of change framework. In no particular order or hierarchy, the first activity included “training on health topics suggested by YAGHRE members and promotional measures from MORU researchers.” This seems to demonstrate a receptiveness of MORU researchers promoting the values of YAGHRE members in learning new health topics (health promotion). As a result of the receptiveness and training, YAGHRE members may have felt a sense of recognition and increased understanding of community’s health concerns, and promotional measures. We considered this output to offer a relevance to our activity and thus accountable to our inputs. The ceiling of accountability in theory of change refers to the high likelihood (strong degree of contributions by the input) of outputs triggered by the inputs (Bemme, 2019; De Silva, Lee et al., 2014; Gooding et al., 2018; Hailemariam et al., 2015; Taplin et al., 2013). Beyond the point of ceiling are longer-term outcomes connected by proposed potential pathways (De Silva, Lee et al., 2014). The overall process of co-designing the health education materials and tools based on the suggestions from YAGHRE members may have garnered a sense of respect towards their priorities ultimately supporting one of the goals of CE.

The second activity entailed observation and interaction of YAGHRE members at MORU’s research laboratory. This opportunity to gain new experience may have increased the familiarity of the laboratory set-up, and the core of the research activities. Such a participatory nature of engagement can enhance building of both formal and informal relationships, transparency (on research process) and ultimately increasing the trust and relationship between the YAGHRE members and the researchers.

The third activity was preparing health education materials (e.g. photos, posters, and videos). The preparation of these health education materials was supported by MORU’s researchers where YAGHRE members developed their skills and capacities to use computers, software, and tools to prepare audio-visual materials. Development of these key skills may have enhanced their understanding of the health topics and the process of research ultimately improving health and research literacy.

The fourth activity was involvement in health campaigns such as COVID-19 vaccination in the district. Such an opportunity to participate in a vaccination campaign can enhance a sense of responsibility and enthusiasm to learn and lead such activities. The responsibility of being involved in the vaccination campaign could have promoted youth group members’ sense of recognition, achievement, and confidence. Beyond the ceiling of accountability, youth group members’ involvement in vaccination campaigns including MORU’s research works may have contributed to the uptake of vaccine and research interventions.

The fifth activity was youth group members’ continuous exploration of community behaviours related to health and the local disease burden. Findings from this formative exploration may have helped direct research and interventions in the community such as tailored (community-informed) health promotional campaigns. Beyond the complete attributability (ceiling of accountability) of such activities, the consequent development of tailored health promotional interventions could promote healthy community behaviour, reducing health burdens and thus can ultimately improve the community’s health.

Brief descriptions of data, objectives and potential outcomes are presented in Table 1. Interpretations of data entailed reiterative reflections by authors and abstraction against the goals/principles underpinning community/youth engagement. At the outset, engagement entailed an orientation meeting with all the stakeholders (district administrative authorities, health authorities, school administration, and MORU researchers). The initial meeting outlined the process of recruitment for school students to become YAGHRE members, followed by engagement activities at MORU’s research station.

YAGHRE members subsequently learnt about the history and scope of MORU’s research in Southeast Asia and in Cambodia including disease priorities. The knowledge and understanding about MORU’s history and research may have contributed to the image and importance of MORU, ultimately supporting the relationship and trust between YAGHRE members, MORU researchers, and community members. YAGHRE members, specifically learnt to use computer, Microsoft office programmes (MS Word, PowerPoint, Excel), camera, drone, and multiple other software applications. The development of these soft skills helped YAGHRE members to prepare audio-visual materials such as PowerPoint slides, posters, and videos. In addition to these skill sets, members also supported the health promotional campaigns including the COVID-19 vaccination programme.

## Results

The overarching outcomes derived from the theory of change (Box 1) were derived from a literature review (Adhikari et al., 2017; Adhikari et al., 2020; Dickert & Sugarman, 2005; Emanuel et al., 2000; Emanuel et al., 2004; Lavery et al., 2010; van Delden & van der Graaf, 2017). Specifically, three outcomes (goals 1, 2, and 4) aligned with the goals of CE for ethical global health research (Adhikari et al., 2020), while two goals (goals 3 and 5) echoed with broader aims of CE for health promotion (Cyril et al., 2015; Harris et al., 2015).

Box 1. **Outcomes of the YAGHRE activities in Siem Pang.**

1. Increased respect towards individuals, communities, and stakeholders
2. Increased trust and relationszhips
3. Improved health and research literacy
4. Improved uptake of health and research interventions
5. Improved community health

### Increased respect towards individuals, communities, and stakeholders

YAGHRE activities were much appreciated by the members and their community. Previous CE activities conducted in this area directly supported specific clinical trials or had a specific focus on increasing literacy around the clinical trials (Callery et al., 2021; Lim et al., 2016; Lim et al., 2017; Nguon et al., 2018). Although the YAGHRE was facilitated by MORU staff, the engagement activities were independent, that is, they were not part of any specific research study. This meant that the YAGHRE activities were shaped by feedback from members, school authorities, district, and provincial authorities. The YAGHRE incorporated the feedback and methodology for engagement suggested by its members and stakeholders, and thus seemed to have demonstrated respect towards the participants and stakeholders of YAGHRE, that included students, their parents, teachers and school authorities, and district authority members.

All YAGHRE activities were decided upon and built based on the feedback from the members. Sometimes, the engagement activities and agenda were only refined and technically supported by our MORU engagement team to ensure the activities were feasible and their objectives were fine-tuned. For instance, the group wanted to learn presentation skills, and our staff supported and guided members by first, offering three laptops to be shared among the 10 students, second, guiding and supporting them to use PowerPoint to prepare slides, and third, refining their slides and presentation skills.

Group members were also asked to list out the health concerns that they were interested in learning about. This exercise gave us a glimpse of the health concerns in the community. At the beginning of 2021, Cambodia was engulfed by a new wave of the COVID-19 pandemic and the group chose to learn and disseminate knowledge about COVID-19. Their suggestions were embraced, and they were asked to share their knowledge and present their idea to the research team. Our team together with support from external experts offered suggestions to strengthen the content of the group’s COVID-19 slides for presentation targeted to their fellow students and families. Adopting their agenda and offering guidance were highly appreciated by the YAGHRE.

### Increased trust, working, and social relationships

Participatory engagement activities offered frequent interactions, communication, and exchange of knowledge, skills, and ideas between members, the research staff, and other local stakeholders. While these interactions were characteristically woven around the participatory activities, they also produced a level of intimacy and social relationship. Building on these relationships, over the months the YAGHRE members became a trusted conduit among researchers, the school, and the wider public. Their role as an interface between MORU, schools, and the community may have contributed a common platform for dialogue and exchange, for example on health topics, research, and engagement strategies, and by virtue of their existing familiarity and trust between these groups, it may have built them as a trusted human infrastructure. Their affiliation with the research team also gave the group a sense of membership to the research organization. As a result of their involvement with MORU on YAGHRE activities and executing engagement under the support and banner of MORU, they may have acquired a sense and degree of representing MORU.

**Figure 4.**
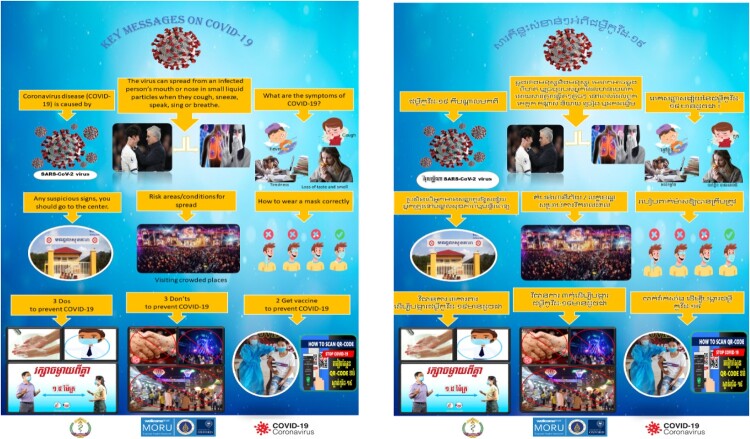
Poster of COVID-19.

The development and implementation of the YAGHRE followed the foremost steps of engagement, by conducting meetings and discussions with all levels of society, including the authorities of the district and province, schools, and other community stakeholders. These orientation meetings at the outset and subsequent meetings where these authority members were invited may have helped to build relationships between MORU staff, YAGHRE members, the authorities, and local stakeholders. Such meetings were also venues for authority members, and stakeholders to share their feedback of the activities. Such feedback and appreciation promoted both MORU staff and youth group members to maintain a regular presence in the health centre and participation in public health activities. One example of how these students developed a working relationship with the authorities and built trust is that the members were asked to help to support the government’s local vaccination campaigns. YAGHRE members assisted through a wide spectrum of activities that included providing vaccine-related messages through leaflets, posters (Figure 4), one-on-one conversations with potential vaccinees, responding to participants’ queries and concerns and asking these participants in turn to invite other community members.

In addition to developing and building relationships and trust through their interactions, members have built close relationships with research staff. Their exposure to the laboratories and staff’s enthusiastic briefing about the studies may have strengthened the confidence of students. This two-way exchange environment was a platform to learn from and advise research scientists. These connections may have been the result of repeated exposure to research over time, and formal and informal times spent together, all of which can eventually contribute to the building of trust.

### Improved health and research literacy

**Figure 5.**
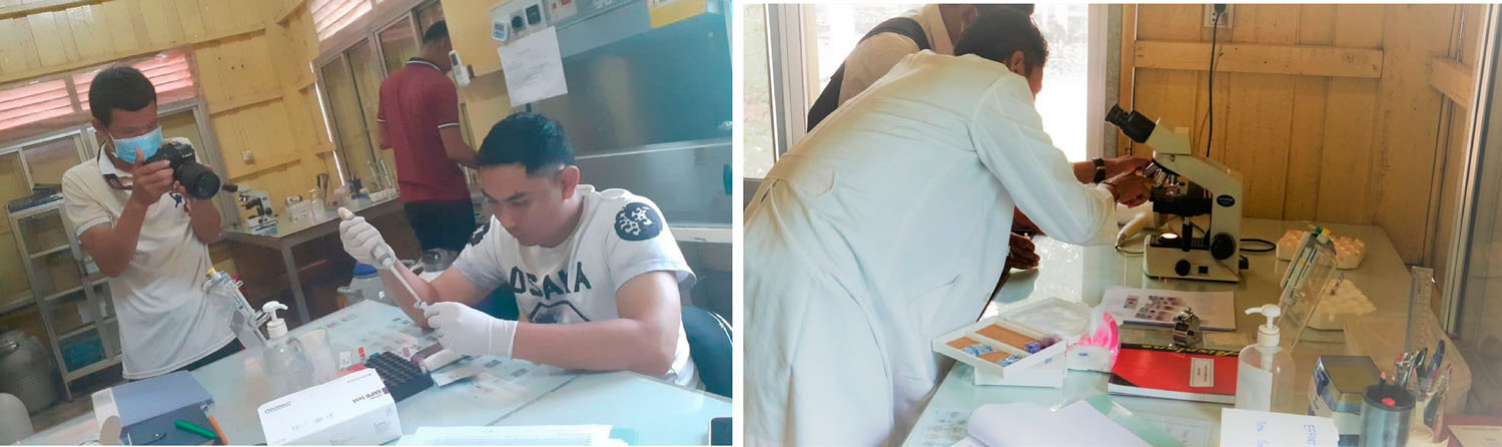
YAGHR members’ visits to the MORU laboratory in Siem Pang.

Engaging YAGHRE members in science and research wherein school students understand the processes and steps of research may have contributed to their confidence around what research is and how health conditions are addressed by research. YAGHRE members have visited MORU’s research facilities where patients are recruited into clinical trials. During such visits, group members have had dialogues about research aims, recruitment processes and the potential outcomes of the research. Members of the YAGHRE have been encouraged to pair up with staff on duty to learn and question about the research and recruitment process including how patients’ laboratory tests are assessed in the lab (Figure 5). In such exposures where YAGHRE members are not just distantly briefed about the lab processes but were allowed to be observers. This may have helped YAGHRE members to understand the inner cores of research processes such as initial assessments, formal screening, recruitment, and how samples move through the laboratory.

**Figure 6.**
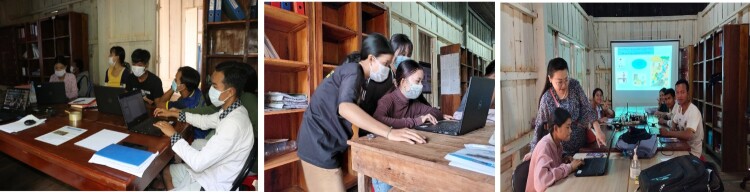
Photo of YAGHR members using computers/laptops.

Participatory engagement with YAGHRE members may also have contributed to the development of specific skill sets and capacity. Members seem to have learnt about the health conditions and research. Over the months, the YAGHRE members have learnt to use Microsoft PowerPoint to create presentations, which they presented to fellow students, parents, and neighbours (Figure 6). Preparing PowerPoint slides on specific topics entailed the selection of the topic or disease, brainstorming on their baseline knowledge, followed by collating information using the internet and consulting CE and content experts. In addition to building their knowledge base, preparing slides for presentations and co-learning with the CE experts, they have also learnt to present their content to their fellow students. In addition, YAGHRE members also learnt to record their presentation using modern software applications such as Zoom and Microsoft Teams. These records have also been shared through a Facebook page (YAGHRE, 2024a) and YouTube (YAGHRE, 2024b) channels they developed for this project. Records of their presentations have been shared widely through these online sources/media. These new skill sets and achievements were deemed to promote YAGHRE members’ confidence and enthusiasm to present on what they have learnt to fellow students and community members and were reflected during feedback sessions with the members.

### Improved uptake of health and research interventions

Youth group members seem to have become a conduit and a connect between the wider public and MORU researchers as well as other healthcare staff. Their understanding of research and interventions was shared with fellow students, parents, and community members. The impact of knowledge sharing to fellow students, parents and neighbours is difficult to assess, particularly because it is complex to determine how much this translated into the uptake of healthy behaviour and interventions (e.g. accepting vaccines). Nonetheless, sharing their knowledge on health topics, research and public health interventions was appreciated by the participants. In addition to sharing knowledge about health and research, YAGHRE members were also directly involved in promoting the uptake of the COVID-19 vaccine as detailed previously.

Heterogeneity in interests among the YAGHRE members also affected their knowledge sharing activities, however, both reactive (when they planned to present) and proactive (spontaneous sharing of knowledge and information) approaches in their knowledge sharing were reported. Regardless of their approaches, scheduled YAGHRE activities covered students in their schools, and the wider community members. Such activities around knowledge sharing may have contributed to expanding the knowledge base, counteracting the potential confusions, rumours, and false perceptions around research conducted locally. The members debriefed the research staff following their health topics engagement activities, commenting on its potential impact, and they shared the opinions and perceptions around health and research from their audiences. YAGHRE members shared the receptivity of the audience, and improvement in knowledge based on their interactions and their efforts in rectifying erroneous beliefs about health and research. Partly, such changes and improvement in knowledge were assessed through informal conversations after the presentations and such reflections were echoed in the pre- and post-presentation questionnaires to serve our own internal evaluation.

Sharing knowledge and health topics engagement from fellow students to their parents and wider community members was encouraged. Specifically, because COVID-19 was a major concern, YAGHRE members also asked fellow students to share the learnt knowledge to their parents and wider community members. Although it is beyond the scope of this study, how much these students shared their knowledge and the coverage, as a part of our self-evaluation, students were encouraged to assess their parents’ knowledge using a brief questionnaire prepared by the youth group (**Supplementary table 1**). Overall, the impact based on the pre- and post- intervention questionnaire was positive. These knowledge sharing activities, and community-wide engagement events undertaken by students may have promoted community members’ knowledge and likely affected health-related behaviours.

### Improved community health

Group members together with research staff discussed the most relevant community health problems and their possible relationship to the community behaviour. Members discussed with their parents and convened to share their findings at the research station. Members shared the priority health problems in their community. Based on the prioritized list of health topics, researchers offered technical and expert knowledge followed by the preparation of health education tools and materials which youth group members subsequently took the lead on and presented in their schools. These also helped MORU researchers more broadly in terms of setting research priorities for the future. Health education materials were presented to community members to ensure that they reached beyond school students. For instance, in 2021, during the COVID-19 pandemic, a total of three presentations were made at three schools that had a total of 159 students; and three presentations in the community with a total of 241 community members. In 2022, YAGHRE expanded the health topics to include first aid training and reached 1140 students from seven schools and 164 community members. These activities were also shared on YAGHRE’s Facebook page (YAGHRE, 2024) and YouTube (YAGHRE, 2024) channel to reach a broader audience. These health education materials may have contributed to the promotion of healthy behaviour and better community health.

Apart from health education materials and the sharing of knowledge with the community members, group members have also supported the local health centres’ health campaigns. For instance, in promoting the need for COVID-19 vaccination, offering support on the ground, such as counselling the potential vaccine recipients, and responding to questions and queries including counteracting rumours around vaccines and science in general. Responding to the priority health problems may have garnered community’s genuine interests on the topic and thus may have promoted preventive behaviours delivered by the YAGHRE members.

## Discussion

In this paper, we report our one year review (from May 2021 to May 2022) of youth advisory group on health and health research engagement in Siem Pang, north-eastern Cambodia. The analysis of YAGHRE activities using the theory of change approach has identified five overlapping outcomes and varies by the extent of tangibility. Outcomes presented in this report are evolving and this initial reflection offers us signposts of the YAGHRE to inform future activities and evaluation initiatives. While this initial reflection of YAGHRE has offered insights into the year one of YAGHRE, a thorough evaluation involving external parties is needed to assess the impacts of the YAGHRE.

Youth advisory groups for health and health research engagement are an important model which serves to achieve the intrinsic goals of CE (Davies et al., 2020; Dickert & Sugarman, 2005; Pratt, 2019). The “intrinsic” goals are independent of whether the community/YAGHRE is ancillary to specific health research. The YAGHRE articulates the intrinsic property of CE. We report a participatory YAGHRE from Cambodia, its relevant goals, and its implications based on the reflective analysis (Ben-Ari & Enosh, 2011; Jasper, 2005; Mortari, 2015).

MORU’s research in Siem Pang is broadly categorized into three types: 1. Clinical trials; 2. Epidemiological studies; and 3. Social sciences and ethics research. CE is embedded within all research. Against the “business as usual approach” wherein CE is deliberately embedded to promote the acceptability of health research/intervention, YAGHRE activities in Siem Pang were deemed to serve community/youth to promote their skills, health/research literacy and ultimately improve community health in the long run (Davies et al., 2019 Reynolds & Sariola, 2018;). MORU’s participatory approach in YAGHRE incorporated topics selected by students, including engagement approaches, and tools which were technically supported by MORU staff. Incorporating the suggestions by YAGHRE members may have promoted their perceived sense of value because it respected their opinions (King et al., 2014). Apart from implications borne by participatory engagement with YAGHRE members of a school, such an act of engagement and inclusivity of their opinion and agenda could demonstrate respect and values to the local population (Adhikari et al., 2020; Holzer et al., 2014; Molyneux & Bull, 2013).

Participatory engagement activities with YAGHRE members and the wider stakeholders were considered to contribute to building rapport and relationships. Within such activities and built relationships, the frequency of interactions, dialogue, and knowledge sharing are recognized to enhance familiarity, a sense of social benefit and trust (Adhikari et al., 2018;Adhikari et al., 2022; Geissler et al., 2008; Kamuya et al., 2013; Molyneux et al., 2005; Sahan et al., 2017; Vincent et al., 2024).

Exposure to topics of science and research is recognized to promote the interest and a positive attitude towards science and scientific institutions (Basu & Barton, 2007; Clark et al., 2016). The most essential aspect of a participatory initiative is the act of participation in the science “process” itself and the degree to which such participation can foster more contextually relevant and mutually beneficial outcomes for everyone involved (Geekiyanage et al., 2020; Shalowitz et al., 2009). Youth group members had regular interactions and opportunities to present among the audience which are likely to enhance confidence, communication & presentation skills, and leadership in promoting health and research messages (Basu & Barton, 2007). Engaging science with the public is part of a broader movement that demonstrates the paradigm shift from deficit to dialogue, from didactic teaching in the classroom to equal contribution in knowledge synthesis ultimately promoting epistemic trust (Shalowitz et al., 2009; Stilgoe et al., 2014). Engagement activities are also reported to enhance the presenters’ (scientists) sense of achievement, presentation skills, and leadership including prospects for their career advancements (Adhikari, Hlaing et al., 2019; Davies et al., 2012). Thus YAGHRE activities are a two-way learning process, where students and researchers learn and build knowledge together (Hopfensperger et al., 2021; Leach et al., 2005). Constructing knowledge together by incorporating local values, living experiences and perceptions echoes with current discourse in global health that values learning from the community (Abimbola, 2021; Abimbola, 2023; Mishra et al., 2022). Subsequent to knowledge synthesis process, outcomes management also resonates as an ethical imperative of CE and aligns with the concept of knowledge democracy (Frenk et al., 2014; Harmon, 2006) and the need for which was prominent during the COVID-19 pandemic (Silva et al., 2021).

New skills learnt by the students are an asset, some of which are likely to be retained for the future that included computer literacy and being able to carry out simple data analysis. Davies et al.’s school engagement in Kenya has demonstrated the variety of impacts on students that ranged from learning new skills (such as photography and participatory video making) to pursuing a career in science (Davies, 2017; Davies et al., 2012). The skills and capacities developed by the YAGHRE members may serve their future career prospects.

Incorporating a community’s own perceptions of health priorities to design health promotion materials is a potential way to improve the community’s health. Addressing community’s concerns, and their priorities also reassures the relevance of the health promotion material (Adhikari et al., 2017; Adhikari et al., 2022; Gittelsohn et al., 2006; Newes-Adeyi et al., 2000; Strachan et al., 2016; Vincent, Adhikari, Duddy et al., 2022). Apart from designing health promotional activities, formative activities carried out to understand the community’s behaviour helps to tailor research needs and public health interventions. The role and contributions of formative research have been found to be critical to successfully tailoring interventions (Adhikari et al., 2017; Strachan et al., 2016).

### Strengths and limitations

The findings were based on the analysis of engagement activity reports, discussions with the CE team, experts and external evaluation consultants. Nonetheless, there are several limitations of this study. The study findings are based on the theory of change framework but were led by MORU’s researchers and therefore may have been biased. Cultural etiquette and power differences may have contributed to the high degree of appreciation and conformism expressed by YAGHRE members, which were predominated by potential benefits and our repeated efforts in exploring the limitations of our engagement activities were futile. Nonetheless, some of the areas for improvement shared were tangential to the current manuscript and were relevant to the overall improvement of engagement that included the need for more dedicated staff to supervise engagement activities, flexibility in the budget to accommodate the changes in engagement activities, strategies, and additional resources. YAGHRE members and engagement coordinators raised issues on the sustainability of engagement efforts particularly referring to the dependence on funding support from external donors. The first year of engagement activities was heavily affected by COVID-19 pandemic, and our activities were focused on supporting the district health centre’s COVID-19 prevention campaigns including vaccination. In the future, YAGHRE will influence MORU's research priorities and design.

## Conclusion

We conclude that the youth advisory group on health and research engagement activities was an effective model of CE so far. We have outlined through a theory of change framework how this was achieved. This project developed a cadre of health and young people who engaged with researchers, fellow students, their families, and the wider community. Although we do not have the quantitative evidence, we believe the YAGHRE activities have contributed to MORU’s overarching engagement outcomes which are: 1. MORU programmes are trustworthy; 2. The potential health impacts of MORU programmes are maximized; and 3. MORU programmes are ethical.

## Supplementary Material

SUPPLEMENTARY FILES.docx
