# Supplementary material for: A youth advisory group on health and health research in rural Cambodia
Source: Glob Bioeth. 2024 Jun 7;35(1):2361968. doi: 10.1080/11287462.2024.2361968 (PMC11164040; doi:10.1080/11287462.2024.2361968)
Supplement: SUPPLEMENTARY FILES.docx [file RGBE_A_2361968_SM3325.docx]

Supplementary **table 1**: Pre- and post-presentation questionnaire

| **Result of Pre- and Post-Test for Students** | | | |  |  |  |  |  |  |  |  |
| --- | --- | --- | --- | --- | --- | --- | --- | --- | --- | --- | --- |
| **Health education session of COVID-19** | | | |  |  |  |  |  |  |  |  |
| Name of School | Total of Student | Result of Pre-Test | | | | | Result of Post-Test | | | | |
|  |  | Poor (<50%) | Average (50%-69%) | Fairly Good 70%-89% | Good 90%-99% | Excellent 100% | Poor (<50%) | Average (50%-69%) | Fairly Good 70%-89% | Good 90%-99% | Excellent 100% |
| Sre Sombo Secondary School | 41 | 2 | 13 | 9 | 17 | 0 | 2 | 5 | 15 | 12 | 7 |
| Prek Meas Secondary School | 59 | 12 | 14 | 16 | 15 | 2 | 8 | 15 | 10 | 16 | 10 |
| Santepheap Secondary School | 59 | 8 | 10 | 24 | 14 | 3 | 5 | 10 | 22 | 12 | 10 |

| **Result of Pre- and Post-Test for family members** |
| --- |
| **Health education session of COVID-19** |

| Name of School | Total questionnaire provided for family member | Total questionnaire received from family member (pre- and post-test) | Result of Pre-Test | | | | | Result of Post-Test | | | | |
| --- | --- | --- | --- | --- | --- | --- | --- | --- | --- | --- | --- | --- |
|  |  |  | Poor (<50%) | Average (50%-69%) | Fairly Good 70%-89% | Good 90%-99% | Excellent 100% | Poor (<50%) | Average (50%-69%) | Fairly Good 70%-89% | Good 90%-99% | Excellent 100% |
| Sre Sombo Secondary School | 210 | 71+71 | 20 | 18 | 14 | 17 | 2 | 1 | 11 | 23 | 24 | 12 |
| Prek Meas Secondary School | 242 | 72+72 | 19 | 3 | 40 | 9 | 1 | 7 | 9 | 23 | 20 | 13 |
| Santepheap Secondary School | 118 | 45+45 | 9 | 12 | 10 | 9 | 5 | 5 | 7 | 11 | 4 | 18 |
